# Imaging inflammation in atherosclerotic plaques, targeting SST_2_ with [^111^In]In-DOTA-JR11

**DOI:** 10.1007/s12350-020-02046-y

**Published:** 2020-02-05

**Authors:** Eric J. Meester, Boudewijn J. Krenning, Erik de Blois, Marion de Jong, Antonius F. W. van der Steen, Monique R. Bernsen, Kim van der Heiden

**Affiliations:** 1 Department of Biomedical Engineering, Thorax Center, Erasmus Medical Center, PO Box 2040, 3000 CA Rotterdam, The Netherlands; 2 Department of Radiology & Nuclear Medicine, Erasmus MC, Rotterdam, The Netherlands; 3 Department of Cardiology, Thorax Center, Erasmus MC, Rotterdam, The Netherlands

**Keywords:** SPECT, atherosclerosis, inflammation, molecular imaging

## Abstract

**Background:**

Imaging Somatostatin Subtype Receptor 2 (SST_2_) expressing macrophages by [DOTA,Tyr^3^]-octreotate (DOTATATE) has proven successful for plaque detection. DOTA-JR11 is a SST_2_ targeting ligand with a five times higher tumor uptake than DOTATATE, and holds promise to improve plaque imaging. The aim of this study was to evaluate the potential of DOTA-JR11 for plaque detection.

**Methods and Results:**

Atherosclerotic ApoE^−/−^ mice (*n* = 22) fed an atherogenic diet were imaged by SPECT/CT two hours post injection of [^111^In]In-DOTA-JR11 (~ 200 pmol, ~ 50 MBq). In vivo plaque uptake of [^111^In]In-DOTA-JR11 was visible in all mice, with a target-to-background-ratio (TBR) of 2.23 ± 0.35. Post-mortem scans after thymectomy and ex vivo scans of the arteries after excision of the arteries confirmed plaque uptake of the radioligand with TBRs of 2.46 ± 0.52 and 3.43 ± 1.45 respectively. Oil red O lipid-staining and ex vivo autoradiography of excised arteries showed [^111^In]In-DOTA-JR11 uptake at plaque locations. Histological processing showed CD68 (macrophages) and SST_2_ expressing cells in plaques. SPECT/CT, in vitro autoradiography and immunohistochemistry performed on slices of a human carotid endarterectomy sample showed [^111^In]In-DOTA-JR11 uptake at plaque locations containing CD68 and SST_2_ expressing cells.

**Conclusions:**

The results of this study indicate DOTA-JR11 as a promising ligand for visualization of atherosclerotic plaque inflammation.

**Electronic supplementary material:**

The online version of this article (doi:10.1007/s12350-020-02046-y) contains supplementary material, which is available to authorized users.

## Introduction

Cardiovascular disease is the leading cause of death worldwide.^1^ Most cardiovascular events are caused by atherosclerosis, in which plaques form over time due to continuous inflammation and lipid deposition in the arterial wall. Current imaging techniques focus on plaque morphology or measures such as calcium score, which are used for cardiovascular risk assessment to improve clinical risk scores.^2^ Inflammation is a crucial factor in atherosclerotic plaque and plays a crucial role in plaque initiation, progression, and destabilization.^3^,^4^ Imaging plaque inflammation may complement traditional imaging methods, providing a better risk stratification of patients at risk of future cardiovascular events.

2-Deoxy-2-[^18^F]fluoro-d-glucose ([^18^F]FDG) has proven a reliable non-invasive imaging method not only to detect, but even to quantify the degree of inflammation in plaques.^5^^–^7^18^F-FDG, therefore, provides a valuable tool to assess and monitor disease burden.^8^ However, background uptake in normal tissue and high uptake of ^18^F-FDG in the myocardium severely hinders detection of coronary plaques,^9^,^10^ and warrants the search for novel radioligands.

Somatostatin Subtype Receptor 2 (SST_2_) is highly expressed on activated macrophages.^11^,^12^ As macrophages are the main inflammatory cell type in atherosclerotic plaque, SST_2_ has been proposed as a relevant imaging target for inflammation-based plaque detection. A number of studies have reported on the use of SST_2_ for inflammation-based imaging of atherosclerosis.^13^^–^19 Moreover, Tarkin et al. recently prospectively validated [^68^Ga]Ga-[DOTA, Tyr^3^]-octreotate (DOTATATE) as a marker for plaque inflammation 20 in a clinical study. They demonstrated the feasibility to detect both carotid and coronary plaques with [^68^Ga]Ga-DOTATATE. Moreover, [^68^Ga]Ga-DOTATATE could better discriminate between high-risk and low-risk lesions compared to [^18^F]FDG.

Various radioligands, based on SST_2_ agonists, for SST_2_-targeted imaging and therapy have been developed and used over the past 20 years for detection and treatment of SST_2_-expressing tumors.^21^ More recently, a new generation of radioligands based on SST_2_ antagonists has been developed and described, showing more favorable pharmacokinetics and higher tumor uptake than agonists like DOTATATE. Of these, the compound JR11 (Cpac[d-Cys-Aph(Hor)-d-Aph(Cbm)-Lys-Thr-Cys]-d-Tyr-NH_2_) performed best in preclinical and clinical studies as an imaging as well as a therapeutic agent.^21^^–^24 Based on the reported favorable biodistribution and targeting efficiency of DOTA-JR11, we studied the potential of DOTA-JR11 in inflammation imaging for atherosclerotic plaque detection as it could yield higher TBRs than agonistic radioligands. We therefore used [^111^In]In-DOTA-JR11 Single Photon Emission Computed Tomography/Computed Tomography (SPECT/CT) to image plaques in vivo in an atherosclerotic mouse model, and assessed target binding in human plaque material.

## Materials and Methods

### Animals and Experimental Setup

Atherosclerotic female ApoE^−/−^ mice on a C67BL/6J background (*n* = 22) were purchased from Charles Rivers (Calco, Italy) at 6 weeks of age, and were fed a high fat diet (0.3% cholesterol, Altromin Spezialfutter GmbH & Co. KG, Lage, Germany) ad libitum from an age of 8 weeks up to 20 weeks. All animal experiments were approved by the institutional animal studies committee and were in accordance with Dutch animal ethical legislation and the European Union Directive.

### Radiolabeling

[^111^In]In-DOTA-JR11 (MW = 1690 g/mol) (kindly provided by Dr. Helmut Maecke) was radiolabeled with [^111^In]InCl_3_ (Covidien, Petten, The Netherlands) with a specific activity of 200 MBq/nmol as described previously.^25^ Radiochemical purity (> 95%) and incorporation yield (> 99%) were evaluated with high-pressure liquid chromatography and instant thin-layer chromatography on silica gel. Quenchers (3.5 mM gentisic acid, 3.5 mM ascorbic acid, 7% ethanol) were added to prevent radiolysis as described previously.^26^

### In Vivo Imaging and Validation

Mice (*n* = 22) were injected with 50 MBq/200 pmol [^111^In]In-DOTA-JR11 in Phosphate Buffered Saline (PBS) with 0.1% Bovine Serum Albumin (BSA), with a total injection volume of 150 µL. Four mice were co-injected with a 100 × excess of unlabeled DOTA-JR11 to test the specificity of the radioligand. Mice were anesthetized with 1.5% isofluorane 2 hours post injection, after which they were injected with 50 µL CT-contrast agent (Exitron Nano 12000, Milteny Biotec, Bergisch-Gladbach, Germany). Immediately after contrast agent injection, mice were transferred to a VECTor5/CT scanner (MILabs B.V. Utrecht, The Netherlands) on which a CT scan was made followed by a SPECT scan. The time between radioligand injection and imaging was based on pilot experiments (data not shown). The CT scan was made with the following settings: full scan angle; accurate scan mode; 50 kV tube voltage; 0.24 mA tube current; 3.5 minute scan time. CT scans were reconstructed at a resolution of 80 µm. SPECT was performed with the M3.0 pinhole collimator (resolution < 1.3 mm, sensitivity > 30,000 cps/MBq). Data were acquired in list-mode using the following acquisition parameters: 1 h scan; 15 positions; spiral scan mode; fine step mode. Scans were reconstructed with energy windows incorporating a width of 20% of the In-111 photo peaks of 171 and 245 keV with background windows of 2.5% on either side of the photo peak windows, and scatter correction was applied according to Ref. 27 Reconstructions were performed with a SROSEM (Similarity Regulated Ordered Subset Expectation Maximization^28^) algorithm with 9 iterations, 128 subsets, with a voxel size of 0.4 mm and a post reconstruction 3-dimensional Gaussian filter was applied (1 mm full width at half maximum).

After in vivo imaging, mice were euthanized with an overdose of isoflurane, after which the vasculature was flushed with PBS via the left ventricle, and thymectomy was performed. In this state, the thorax of the euthanized mice was scanned ‘in situ’ to circumvent signal interference from thymic uptake of [^111^In]In-DOTA-JR11 with signal from plaque uptake. CT and SPECT settings for in situ imaging were the same as for in vivo imaging, except for a shorter SPECT scan duration of 30 minutes.

The arteries were removed after in situ imaging, and cleaned of remaining connective tissue. They were then stained for lipids (Oil Red O (ORO) according to standard protocol) to confirm plaque presence, and scanned ex vivo with SPECT/CT. The scan settings for ex vivo imaging were the same as in situ settings except for four SPECT positions due to a smaller field of view. Subsequently, the arteries were cut open and used for *ex vivo* autoradiography (*n* = 4) or embedded in Tissue-Tek O.C.T. compound (Sakura Finetek Europe B.V., Alphen aan den Rijn, The Netherlands) and stored at – 80 °C for histological analysis (*n* = 14). After ~ 2 weeks, arteries used for ex vivo autoradiography were placed on a phosphor screen overnight and read using a phosphor imager (Cyclone, Perkin Elmer).

### Ex vivo Carotid Endarterectomy Study

To investigate binding of [^111^In]In-DOTA-JR11 to and its potential for imaging of human plaque tissue, we performed an *ex vivo* study with human carotid endarterectomy (CEA) tissue slices. For this purpose we sliced a CEA sample (acquired with informed consent and approved by the medical ethics committee of the Erasmus MC, MEC 2008-147) into 2 mm slices. Even-numbered slices were embedded in O.C.T. compound and stored at − 80° C for later in vitro binding assays and histological evaluation. Odd-numbered slices were incubated with 200 MBq/1 nmol [^111^In]In-DOTA-JR11 in 20 mL PBS with 0.1% BSA for one hour. After incubation, the slices were washed in PBS with 0.1% BSA until no residual radioactivity remained in the washing medium as measured by a dose calibrator (Dose calibrator VDC-405, Comecer Netherlands, Joure, The Netherlands). The slices were subsequently placed on a holder, and imaged with the VECTor5/CT. CT and SPECT settings were the same as for the in vivo mouse scan, except a mouse 1.6 pinhole collimator (resolution < 1.6 mm, sensitivity > 1500 cps/MBq) was used with 38 scan positions, and the scans were reconstructed with a Gaussian filter of 0.5 mm full width at half maximum.

### Immunohistochemistry and In Vitro Binding Assays

Embedded mouse arteries and CEA slices were sectioned into 5 µm sections, which were immunohistochemically stained for CD68 (Mouse: 1:100, Biorad, MCA1848; human: 1:100, Abcam, ab955) and SST_2_ (1:100, Abcam, clone UMB-1) to assess target presence and presence of macrophages. In short, sections were fixed in cold acetone for 5 minutes, endogenous peroxidase was blocked with 0.3% H_2_O_2_, and non-specific binding was blocked with 1% BSA for mouse CD68 staining and 2% normal goat serum for SST_2_ and human CD68 staining. The primary antibody was omitted from the protocol in negative controls.

10 µm sections, adjacent to the 5 µm sections used for immunohistochemistry, were cut from the embedded CEA slices to assess radioligand uptake via an in vitro competition binding assay (autoradiography). Sections were incubated for 1 h with 10^−9^ M [^111^In]In-DOTA-JR11 with or without an excess of 10^−6^ M unlabeled DOTA-JR11 to asses specific binding. Slides were exposed to phosphor screens overnight and read with a phosphor imager (Cyclone, Perkin Elmer). Haematoxylin–eosin staining according to standard protocol was performed on the sections afterwards.

### Quantification and Statistics

SPECT/CT data were analyzed with Vivoquant (Invicro) by quantification of the activity within manually drawn regions of interests (ROIs) based on contrast-enhanced CT images. In vivo ROIs were drawn for the aortic arch and the brachiocephalic artery, whereas the vena cava inferior and jugular vein were used as background regions. In situ ROIs were the aortic arch and brachiocephalic artery, and the heart ventricles were taken as background tissue. ROIs on ex vivo images were also the aortic arch and brachiocephalic artery for plaque areas, and the relatively healthy descending thoracic aorta as background. Target-to-background-ratios (TBRs) were calculated and expressed as mean ± standard deviation.

In vitro autoradiography analysis was performed by drawing ROIs around tissue sections in Optiquant (Perkin Elmer), quantifying the signal as Digital Light Units (DLU)/mm^2^ and comparing non-blocked to blocked tissue sections.

The Shapiro–Wilk test was used to test data for normality. The student’s *t* test was used to compare means of normally distributed data, the Mann–Whitney *U* test was used for non-parametric data.

## Results

### Mouse Plaque Imaging

**Fig. 1:**
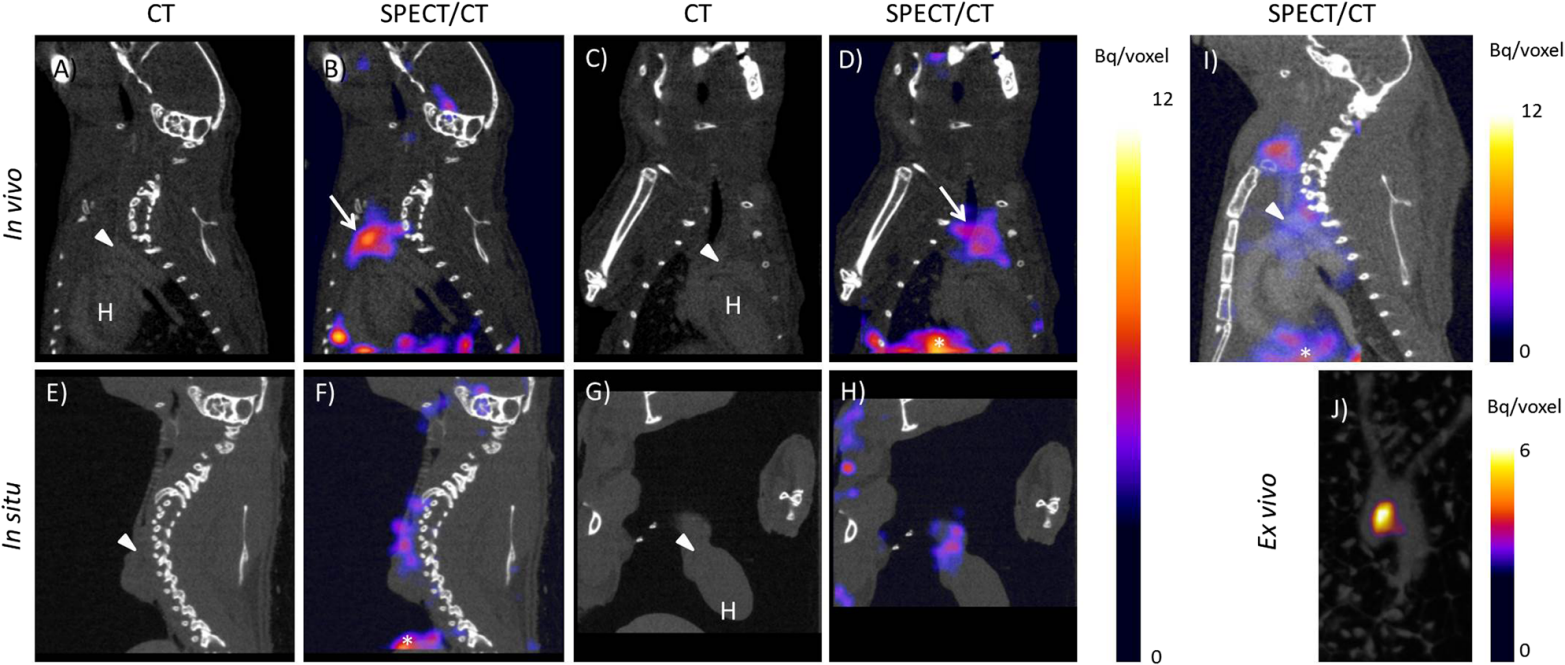
[^111^In]In-DOTA-JR11 uptake in mouse atherosclerotic plaque two hours post injection of 200 pmol [^111^In]In-DOTA-JR11. **A** Sagittal CT, **B** saggital SPECT/CT, **C** coronal CT, and **D** coronal SPECT/CT image of [^111^In]In-DOTA-JR11 uptake *in vivo* in an atherosclerotic mouse. **E** Sagittal CT, **F** saggital SPECT/CT, **G** coronal CT, and **H** coronal SPECT/CT image of [^111^In]In-DOTA-JR11 uptake *in situ* in the mouse displayed in (**A**-**C**) scanned post-mortem after thymectomy and flushing of the vasculature with PBS. **I** Sagittal SPECT/CT image of a mouse two hours post injection of 50 MBq/200 pmol [^111^In]In-DOTA-JR11 plus a 100 × excess of unlabeled DOTA-JR11. Plaque uptake was strongly reduced by blocking. **J** Maximum intensity projection image of the excised arteries of the mouse shown in (**A**-**H**). Focal uptake of [^111^In]In-DOTA-JR11 is visible at the plaque location. Arrowheads indicates the location of the aortic arch containing plaque, arrows indicates thymic uptake of [^111^In]In-DOTA-JR11, *Indicates uptake in the liver, H indicates the heart

In vivo SPECT/CT imaging 2 h after intravenous injection of [^111^In]In-DOTA-JR11 showed focal uptake at locations of plaque formation in the vasculature of all animals (Fig. 1A-D), with an average TBR of 2.23 ± 0.35. Thymic uptake (average TBR of 2.28 ± 0.51) of [^111^In]In-DOTA-JR11 masked plaque signal and therefore complicated visualization and quantification. Therefore, ‘in situ’ scans were made in post-mortem thymectomized animals. In situ SPECT/CT imaging confirmed plaque uptake of [^111^In]In-DOTA-JR11 as seen in in vivo images (Fig. 1E-H), with a TBR of 2.46 ± 0.52. Blocking studies with an 100x excess of DOTA-JR11 significantly reduced the arterial signal (TBR in vivo blocked: 1.47 ± 0.36; TBR in situ blocked: 1.36 ± 0.15, *P* = 0.05, see Fig. 1I and Online Resource 1) Likewise, blocking significantly reduced uptake in the thymus (TBR 1.32 ± 0.43, *P* = 0.05).

**Fig. 2:**
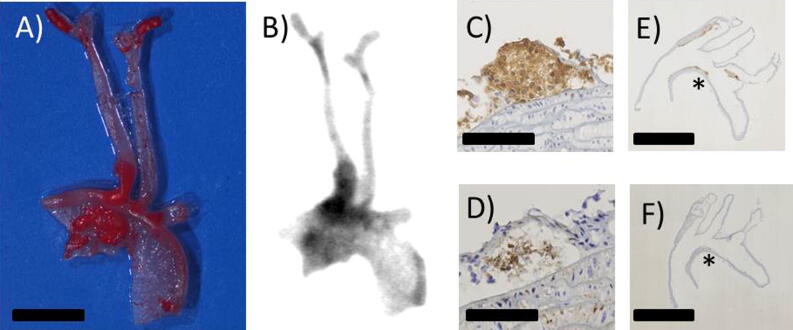
**A** Excised, cut open and Oil red O stained arteries of a mouse injected with 200 pmol [^111^In]In-DOTA-JR11. Scale bar indicates 2 mm. **B** Matching high resolution ex vivo autoradiogram to the arteries shown in (**A**), showing [^111^In]In-DOTA-JR11 uptake at plaque locations. **C**, **D** show immunohistochemistry for CD68 (macrophages) and Somatostatin Subtype Receptor 2 (SST_2_) expressing cells in mouse plaque, respectively. Scale bar indicates 100 µm. **E**, **F** show the overview of the histological sections shown in (**C**) and (**D**); the asterisk marks the location of the zoomed area in (**C**) and (**D**). Scale bar indicates 2.5 mm

Presence of plaque in excised arteries was confirmed by ORO staining of excised arteries. *Ex vivo* SPECT/CT imaging of the mouse arteries showed uptake of [^111^In]In-DOTA-JR11 at plaque locations in the aortic arch and brachiocephalic artery with a TBR of 3.43 ± 1.45 (Fig. 1J). Ex vivo autoradiography and ORO staining of excised, cut open arteries confirmed uptake of [^111^In]In-DOTA-JR11 in plaque (Fig. 2A, B). Immunohistochemistry of the arteries confirmed presence of SST_2_ and CD68 expressing cells in plaque (Fig. 2C-F).

### Human Plaque Imaging

**Fig. 3:**
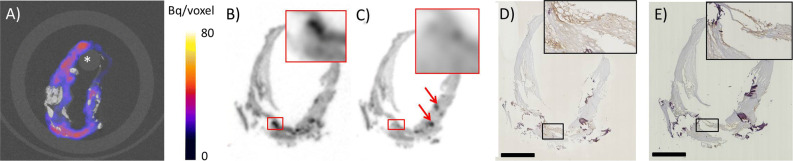
[^111^In]In-DOTA-JR11 uptake in human carotid endarterectomy (CEA) tissue after incubation with [^111^In]In-DOTA-JR11. **A** Transverse SPECT/CT image of a 2 mm slice of a CEA sample. Calcifications are visible in CT in white, the asterisk indicates the holder used to keep the 2 mm slice in place. **B**, **C** In vitro autoradiography of adjacent 10 µm sections made of an adjacent 2 mm slice of the same CEA sample seen in (**A**). The section in **B** was incubated with 10^−9^ M [^111^In]In-DOTA-JR11, the section in **C** was incubated with 10^−9^ M [^111^In]In-DOTA-JR11 plus a blocking dose of 10^−6^ M unlabeled DOTA-JR11. The inset shows the boxed region at higher magnification, the red arrows indicate sectioning artifacts (tissue folds). **D** SST_2_ and **E** CD68 immunohistochemistry on 5 µm sections adjacent to **B** and **C**, with matching insets. Scale bar indicates 2 mm

Two millimeter thick slices of a human carotid endarterectomy sample incubated with [^111^In]In-DOTA-JR11 showed focal hotspots of radioligand uptake detectable by SPECT, reflecting the presence of SST_2_ as determined by immunohistochemistry (Fig. 3). Noticeably, no radioligand uptake or SST_2_ expression was visible in areas of macrocalcifications visible in CT. In vitro autoradiography performed on 10 µm sections of adjacent 2 mm slices showed specific binding of [^111^In]In-DOTA-JR11 when compared to adjacent sections incubated with a 1000 × excess of unlabeled DOTA-JR11 (non-blocked = 35 × 10^6^ ± 90 × 10^5^, blocked = 25 × 10^6^ ± 63 × 10^5^ DLU/mm^2^, *P* = 0.005, see Online Resource 2 and Fig. 3). Moreover, radioligand uptake visualized on in vitro autoradiography co-localized with CD68 and SST_2_ expression on adjacent sections, and signal as seen on the SPECT/CT images of the adjacent 2 mm slices (Fig. 3B-E).

## Discussion

We have demonstrated the feasibility of imaging atherosclerotic plaques by targeting SST_2_ with DOTA-JR11, by visualizing plaque with In-111 labeled DOTA-JR11 in a mouse model of atherosclerosis and in human plaque tissue. We showed that radioligand uptake is located in plaque regions in the mouse vasculature as evidenced by *in vivo* and in situ SPECT/CT imaging, autoradiography, and ORO staining. [^111^In]In-DOTA-JR11 uptake in human plaque tissue co-localizes with SST_2_ and CD68 expressing cells, whereas blocking studies show target-specific uptake in vivo and in situ in mouse plaque, and in vitro in human tissue.

High thymic uptake of [^111^In]In-DOTA-JR11, visible in the in vivo SPECT images and in line with reported SST_2_ expression in mice in this tissue,^29^ is problematic when visualizing plaque in the used atherosclerotic model. Nevertheless, we demonstrated that the in vivo signal visible next to the thymus did originate from plaque, by comparing the intensity and the localization of plaque signal in the in vivo, in situ, and ex vivo SPECT scans, as well as the ex vivo autoradiography. Thymic uptake is not expected to interfere with human plaque imaging as thymic activity wanes during adolescence, and little SST_2_-ligand binding has been found in dedicated studies.^30^ Ex vivo imaging of a human CEA sample showed that SPECT imaging using [^111^In]In-DOTA-JR11 is feasible in human plaque tissue and that radioligand uptake co-localizes to regions of inflammation as evidenced by CD68 and SST_2_ immunohistochemistry. These results indicate that DOTA-JR11 has potential for imaging of inflammation in human plaques.

Oncological studies reported a five times higher uptake of DOTA-JR11 over DOTATATE in SST_2_ positive tumors.^21^^–^23 It is hypothesized that antagonistic ligands such as DOTA-JR11 have more binding sites on the receptor than agonistic ligands such as DOTATATE.^31^ Although the exact mechanism for this difference in uptake remains to be elucidated, the growing amount of studies using SST_2_-mediated imaging in atherosclerosis 13^–^19 indicate DOTA-JR11 as an interesting candidate for further studies.

DOTATATE and DOTA-JR11 have not been compared for the detection of atherosclerotic plaque. Two preclinical studies have tested [^68^Ga]Ga-DOTATATE for plaque imaging in mouse models, however. Although it is difficult to compare studies using different animal models and different imaging systems, the results found in 14,15 indicate that [^68^Ga]Ga-DOTATATE has a lower TBR compared to [^111^In]In-DOTA-JR11 in our study. Rinne et al. found an aorta to blood ratio of 0.67 ± 0.04 using [^68^Ga]Ga-DOTATATE *in vivo* in IFG-II/LDLR^−/−^ApoB^100/100^ mice,^15^ indicating low radioligand uptake in plaque. However, they also reported a high plaque to wall ratio of 2.1 ± 0.5 for [^68^Ga]Ga-DOTATATE in autoradiographic studies. Li et al. found a similar plaque to non plaque ratio of ~ 1.8 after autoradiographic analysis of ApoE^−/−^ arteries incubated with [^68^Ga]Ga-DOTATATE.^14^ We found an in vivo TBR of 2.23 ± 0.35 and an in situ TBR of 2.46 ± 0.52, and in the autoradiogram of the mouse arteries we found a TBR of 3.43 ± 1.45. Taken together, these studies warrant further investigations into the added value of DOTA-JR11 over DOTATATE in atherosclerotic patients.

If a five times higher uptake of DOTA-JR11, as was found in oncological studies, would be found in atherosclerosis as well, DOTA-JR11 could offer possibilities for visualization of less inflamed plaques or plaques with lower SST_2_ expression. Moreover, the DOTA chelator of JR11 allows labeling with different radionuclides including Ga-68, making DOTA-JR11 attractive for PET imaging. Although different radiometals result in differences in binding affinity of DOTA-JR11,^32^ the attractive pharmacokinetics of DOTA-JR11 are conserved when labeled with Ga-68.^24^

Because inflammation in different plaque regions can have a substantial effect on the rupture risk of atherosclerotic plaques, future studies should investigate whether DOTA-JR11 uptake can be correlated to different plaque phenotypes. A better interpretation of radioligand uptake related to plaque phenotype could be a major step in patient risk stratification.

## Conclusion

Our results indicate DOTA-JR11 as a promising ligand for atherosclerosis imaging based on our promising in vivo results and ex vivo validation studies. DOTA-JR11 could be a valuable improvement in imaging of inflammation in atherosclerotic disease.

## New Knowledge Gained

The SST_2_ targeting radioligand [^111^In]In-DOTA-JR11 can be used to detect plaques in a mouse model of atherosclerosis by visualizing plaque inflammation. [^111^In]In-DOTA-JR11 uptake in human plaque tissue indicates the translational potential of this radioligand for human imaging. Recent success of SST_2_ imaging in atherosclerosis with DOTATATE, and a five times higher TBR of DOTA-JR11 than DOTATATE in oncological studies, make DOTA-JR11 an interesting ligand for further studies in atherosclerosis.

## Electronic supplementary material

Below is the link to the electronic supplementary material.

Electronic supplementary material 1 (PDF 711 kb)

Electronic supplementary material 2 (M4A 5634 kb)

Electronic supplementary material 3 (PPTX 2418 kb)

## Abbreviations

SST_2_: Somatostatin subtype receptor 2
SPECT: Single photon emission tomography
CT: Computed tomography
ORO: Oil red O
CEA: Carotid endarterectomy
TBR: Target-to-background-ratio
