# Supplementary material for: Imaging inflammation in atherosclerotic plaques, targeting SST2 with [111In]In-DOTA-JR11
Source: J Nucl Cardiol. 2020 Feb 5;28(6):2506–13. doi: 10.1007/s12350-020-02046-y (PMC8709817; doi:10.1007/s12350-020-02046-y)
Supplement: Supplementary file 1 — Electronic supplementary material 1 (PDF 711 kb) [file 12350_2020_2046_MOESM1_ESM.pdf]

Supplemental material

Imaging inflammation in atherosclerotic plaques, targeting SST_2 with $[^{111}In]In$-DOTA-JR11

Journal of Nuclear Cardiology

Eric J. Meester, MSc[1,2], Boudewijn J. Krenning, MD, PHD, PhD[3], Erik de Blois, PhD[2], Marion de Jong, PhD[2] , Antonius F.W. van der Steen, PhD[1], Monique R. Bernsen, PhD[2]*, Kim van der Heiden, PhD[1]*

1 Department of Biomedical Engineering, ThoraxCenter, Erasmus MC, Rotterdam, The Netherlands

2 Department of Radiology & Nuclear Medicine, Erasmus MC, Rotterdam, The Netherlands

3 Department of Cardiology, ThoraxCenter, Erasmus MC, Rotterdam, The Netherlands

* These authors contributed equally to this work

Correspondence address: Dr. Kim van der Heiden, Department of Biomedical Engineering, Erasmus Medical Center, PO Box 2040, 3000 CA Rotterdam, The Netherlands

E-mail: k.vanderheiden@erasmusmc.nl

Telephone: +31 (0)10 7044045

Fax: +31 (0)10 7044720

Supplemental Figures

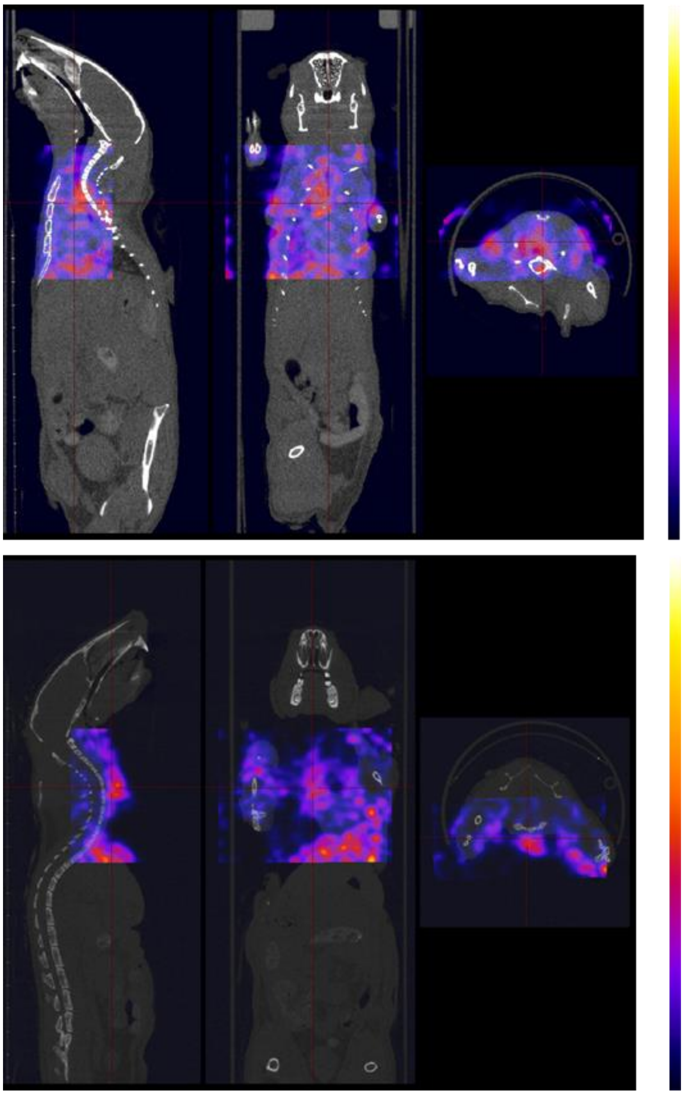

Online Resource 1

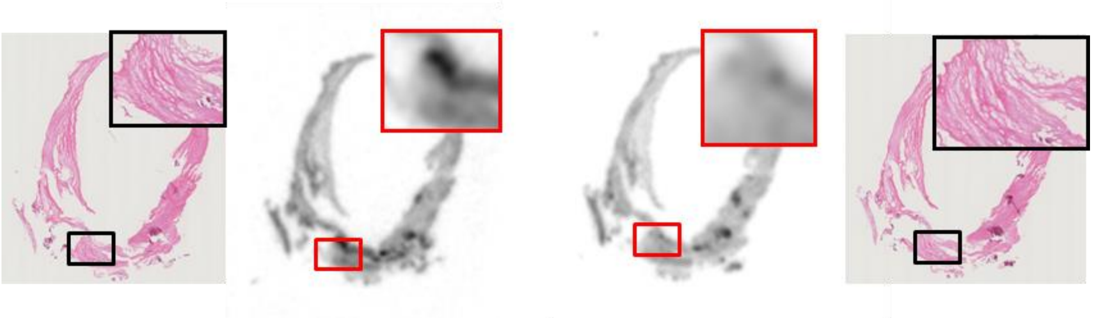

Online Resource 2

Supplemental Figure Legends

Online Resource 1: The upper panel shows an *in vivo* SPECT/CT image in sagittal, coronal, and transverse view of a mouse two hours post injection of 50 MBq/200 pmol [^{111}In]In-DOTA-JR11 plus a 100x excess of unlabelled DOTA-JR11. The plaque location is indicated by the crosshair. Plaque uptake was significantly reduced by blocking (target to background ratio (TBR) *in vivo*: 2.23 ± 0.35; TBR *in vivo* blocked: 1.47 ± 0.36; p=0.05). The lower panel shows the same mouse as in the upper panel after thymectomy. Here the crosshair also indicates the location of the aortic arch. Plaque uptake is significantly reduced by blocking (TBR *in situ*: 2.46 ± 0.52; TBR *in situ* blocked: 1.36 ± 0.15; p=0.05). Note that the images are differently scaled compared to the images in Figure 1, to appreciate the reduction of plaque signal to background levels following blocking.

Online Resource 2: Shows two autoradiograms of adjacent 10 µm sections of a human carotid endarterectomy sample, and the corresponding Haematoxylin-Eosin stained sections. The left sample was incubated for 1 hour with 10^{-9} M [^{111}In]In-DOTA-JR11, whereas the right sample was incubated with 10^{-9} M [^{111}In]In-DOTA-JR11 plus a blocking dose of 10^{-6} M unlabelled DOTA-JR11. Boxed regions show areas of Somatostatin subtype receptor 2 expression in the blocked and unblocked section. Digital light units (DLU) per section were significantly reduced by blocking (non blocked=$35*10^6$±$90*10^5$, blocked=$25*10^6$ ±$63*10^5$ DLU/mm^2, p=0.005, n=7).
