# Supplementary material for: Imaging inflammation in atherosclerotic plaques, targeting SST2 with [111In]In-DOTA-JR11
Source: J Nucl Cardiol. 2020 Feb 5;28(6):2506–13. doi: 10.1007/s12350-020-02046-y (PMC8709817; doi:10.1007/s12350-020-02046-y)
Supplement: Supplementary file 3 — Electronic supplementary material 3 (PPTX 2418 kb) [file 12350_2020_2046_MOESM3_ESM.pptx]

## Slide 1
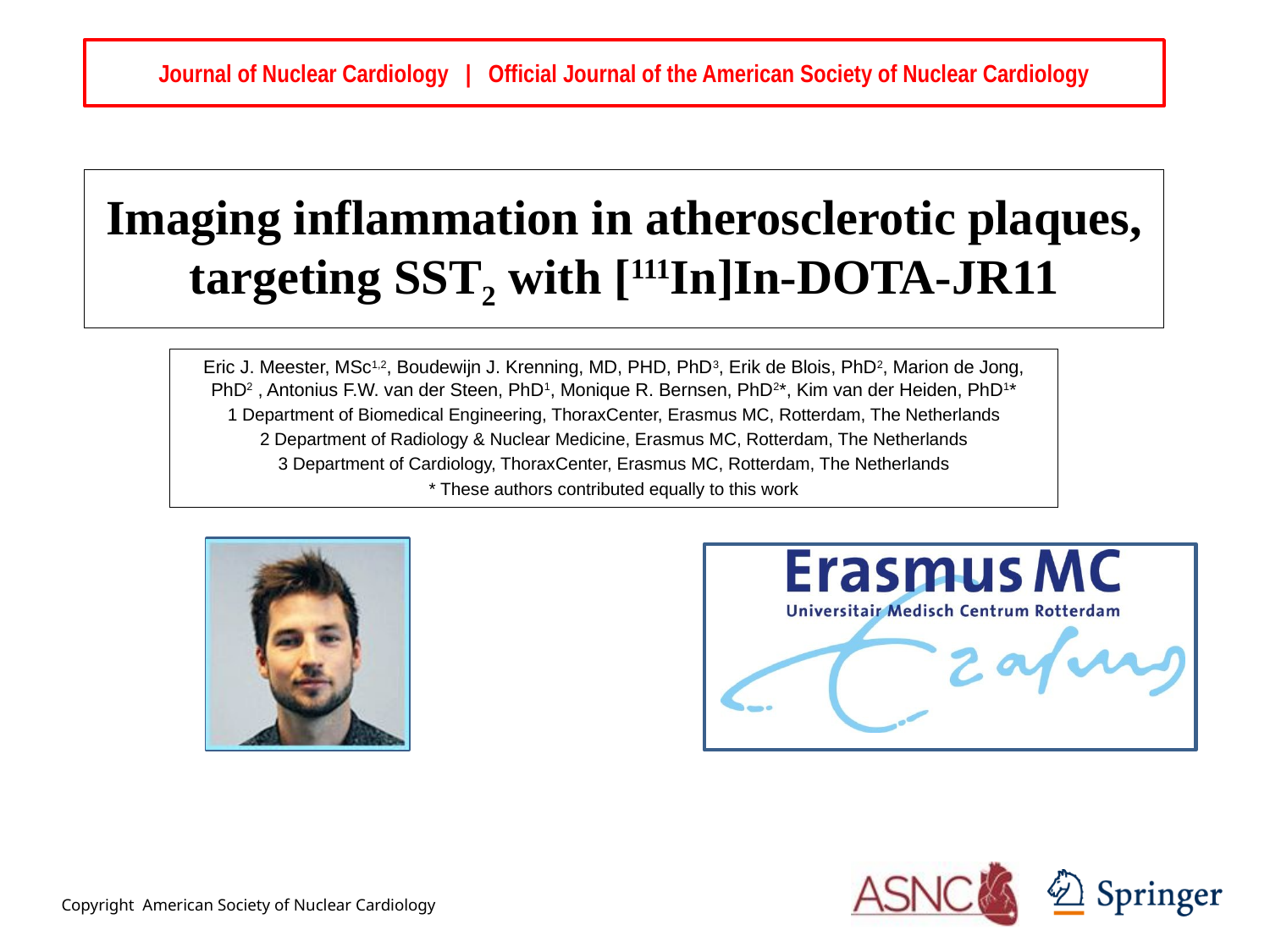

Journal of Nuclear Cardiology | Official Journal of the American Society of Nuclear Cardiology
# Imaging inflammation in atherosclerotic plaques, targeting SST2 with [111In]In-DOTA-JR11
Eric J. Meester, MSc1,2, Boudewijn J. Krenning, MD, PHD, PhD3, Erik de Blois, PhD2, Marion de Jong, PhD2 , Antonius F.W. van der Steen, PhD1, Monique R. Bernsen, PhD2*, Kim van der Heiden, PhD1*
1 Department of Biomedical Engineering, ThoraxCenter, Erasmus MC, Rotterdam, The Netherlands
2 Department of Radiology & Nuclear Medicine, Erasmus MC, Rotterdam, The Netherlands
3 Department of Cardiology, ThoraxCenter, Erasmus MC, Rotterdam, The Netherlands
* These authors contributed equally to this work
Copyright American Society of Nuclear Cardiology

## Slide 2
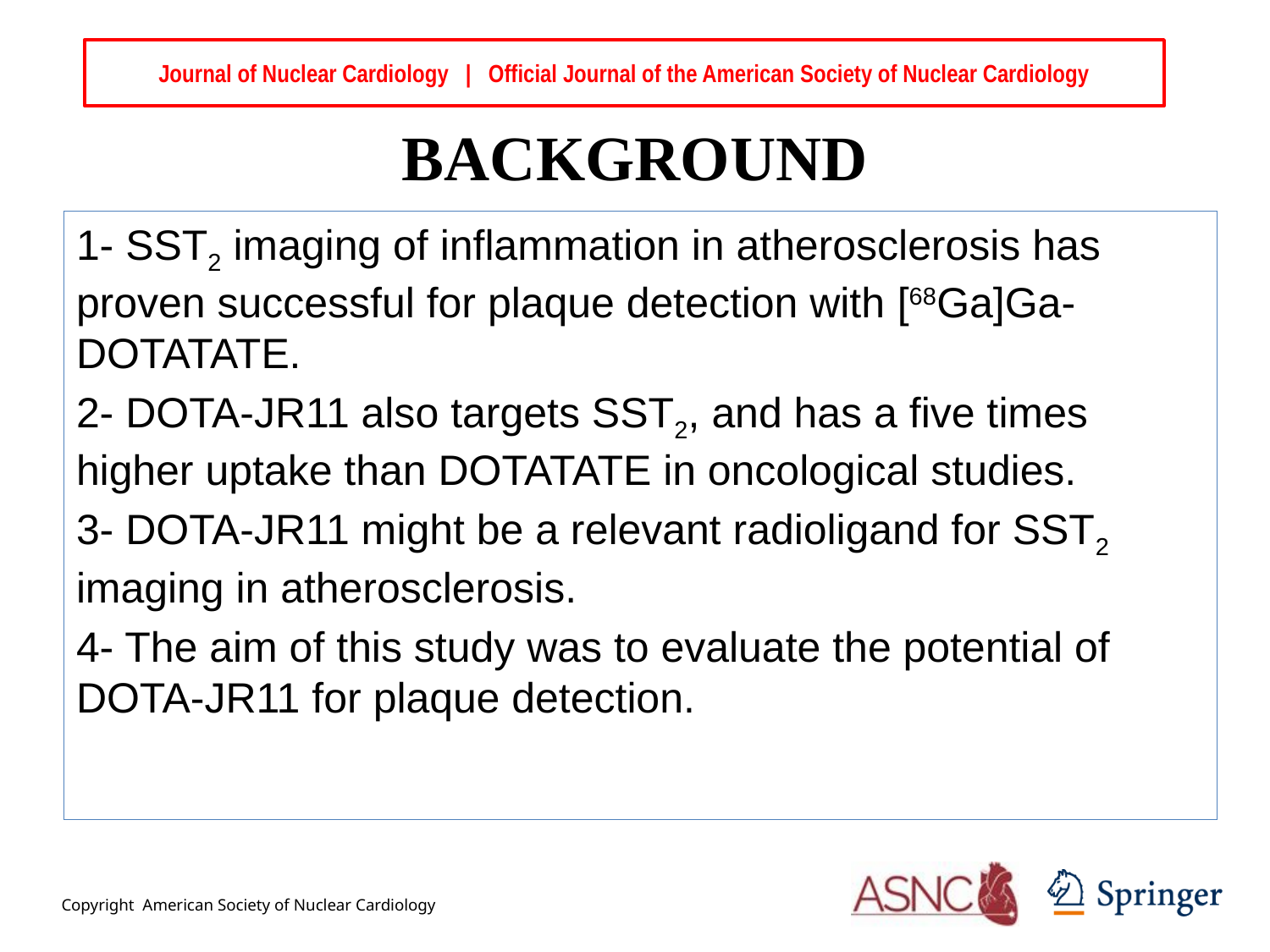

Journal of Nuclear Cardiology | Official Journal of the American Society of Nuclear Cardiology
# BACKGROUND
1- SST2 imaging of inflammation in atherosclerosis has proven successful for plaque detection with [68Ga]Ga-DOTATATE.
2- DOTA-JR11 also targets SST2, and has a five times higher uptake than DOTATATE in oncological studies.
3- DOTA-JR11 might be a relevant radioligand for SST2 imaging in atherosclerosis.
4- The aim of this study was to evaluate the potential of DOTA-JR11 for plaque detection.
Copyright American Society of Nuclear Cardiology

## Slide 3
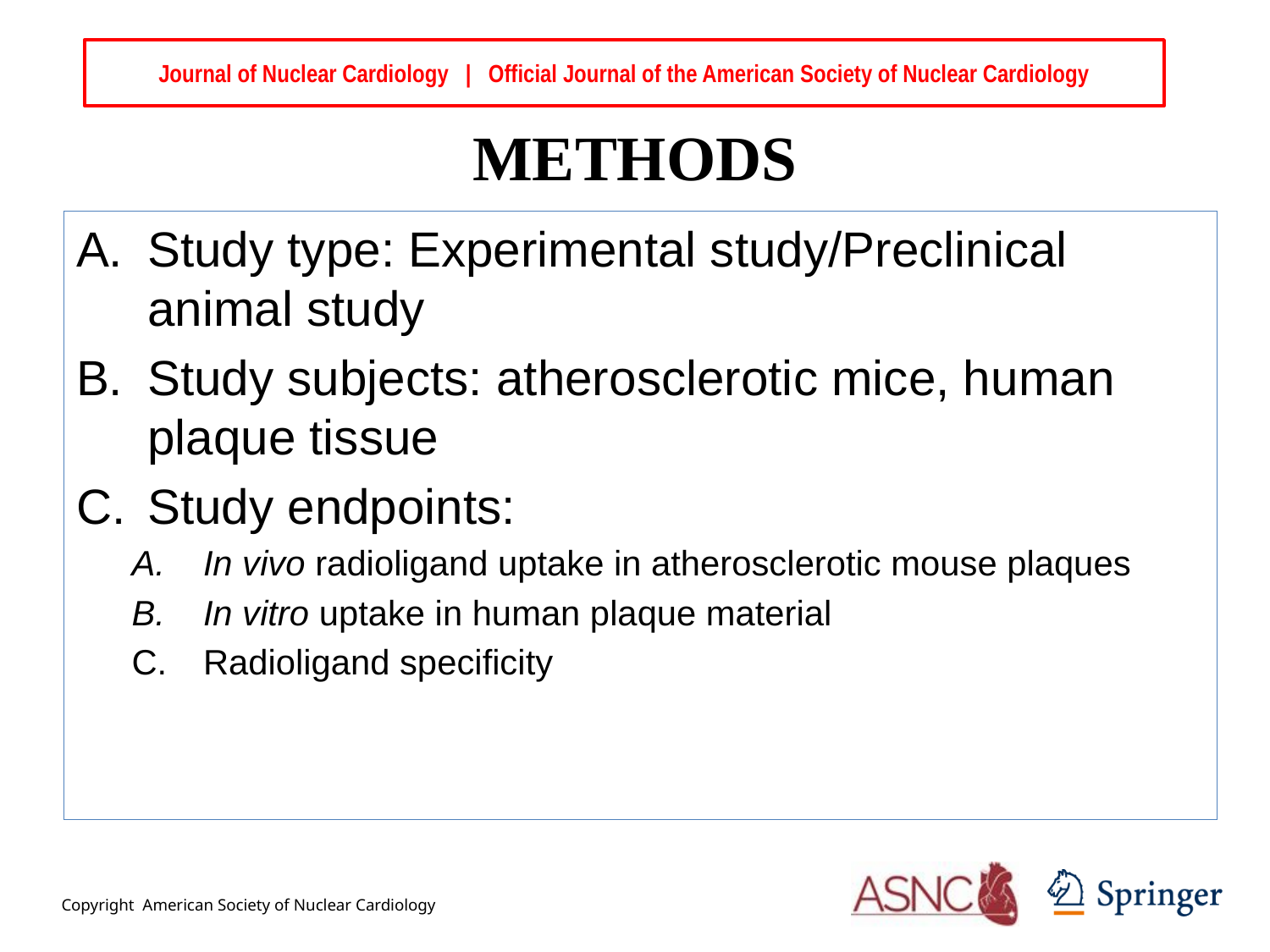

Journal of Nuclear Cardiology | Official Journal of the American Society of Nuclear Cardiology
# METHODS
Study type: Experimental study/Preclinical animal study
Study subjects: atherosclerotic mice, human plaque tissue
Study endpoints:
In vivo radioligand uptake in atherosclerotic mouse plaques
In vitro uptake in human plaque material
Radioligand specificity
Copyright American Society of Nuclear Cardiology

## Slide 4
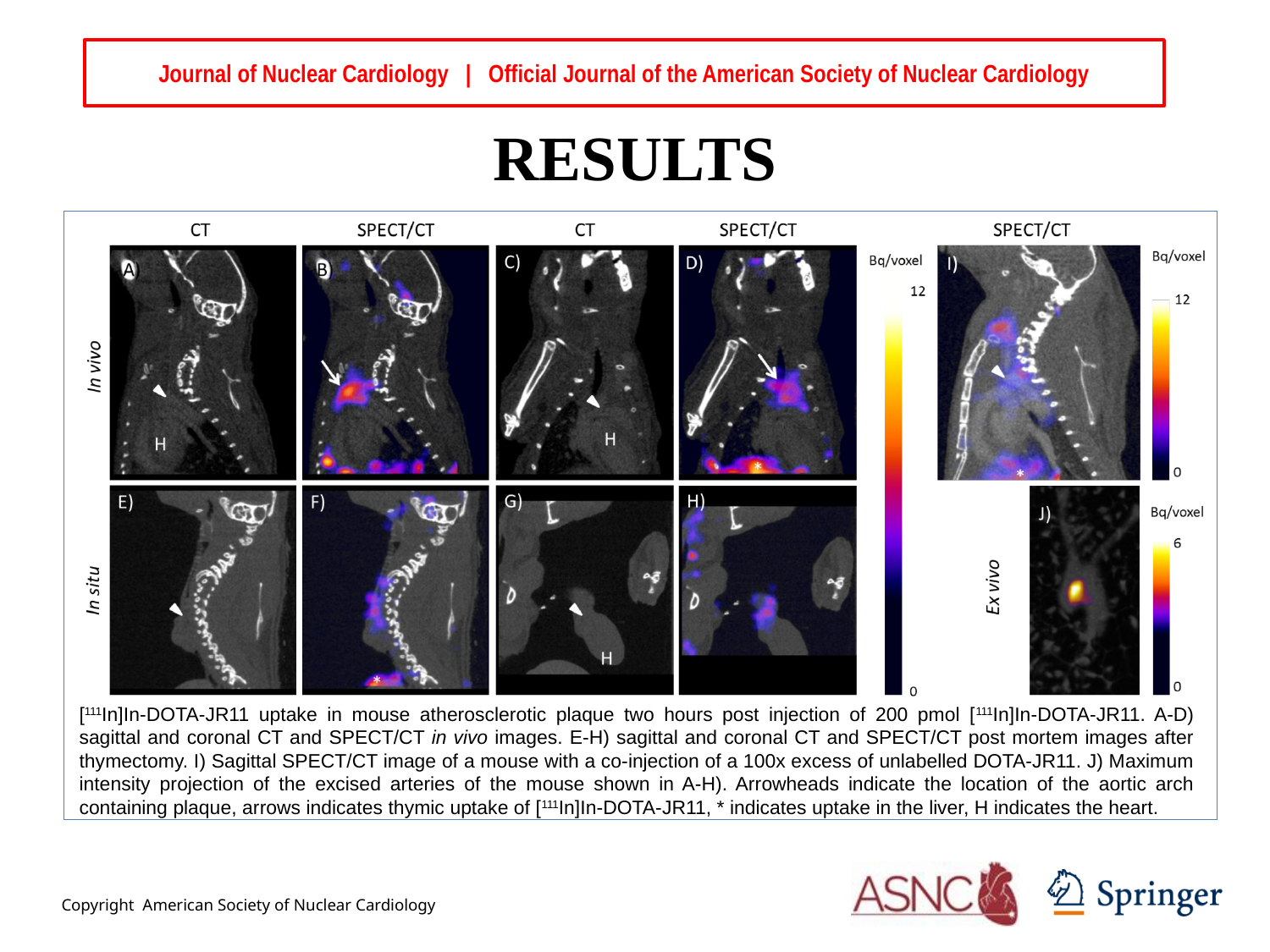

Journal of Nuclear Cardiology | Official Journal of the American Society of Nuclear Cardiology
# RESULTS
[111In]In-DOTA-JR11 uptake in mouse atherosclerotic plaque two hours post injection of 200 pmol [111In]In-DOTA-JR11. A-D) sagittal and coronal CT and SPECT/CT in vivo images. E-H) sagittal and coronal CT and SPECT/CT post mortem images after thymectomy. I) Sagittal SPECT/CT image of a mouse with a co-injection of a 100x excess of unlabelled DOTA-JR11. J) Maximum intensity projection of the excised arteries of the mouse shown in A-H). Arrowheads indicate the location of the aortic arch containing plaque, arrows indicates thymic uptake of [111In]In-DOTA-JR11, * indicates uptake in the liver, H indicates the heart.
Copyright American Society of Nuclear Cardiology

## Slide 5
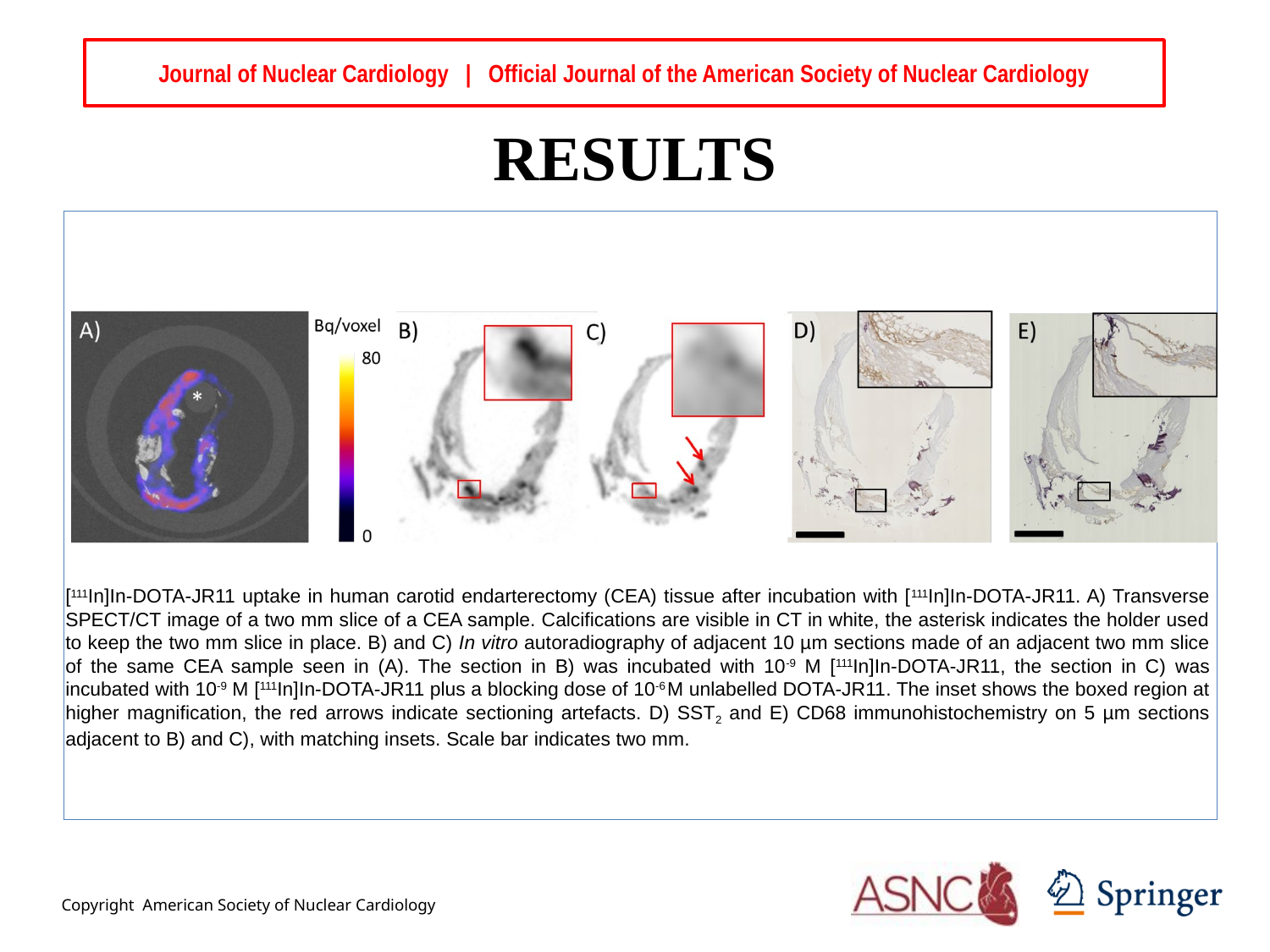

Journal of Nuclear Cardiology | Official Journal of the American Society of Nuclear Cardiology
# RESULTS
[111In]In-DOTA-JR11 uptake in human carotid endarterectomy (CEA) tissue after incubation with [111In]In-DOTA-JR11. A) Transverse SPECT/CT image of a two mm slice of a CEA sample. Calcifications are visible in CT in white, the asterisk indicates the holder used to keep the two mm slice in place. B) and C) In vitro autoradiography of adjacent 10 µm sections made of an adjacent two mm slice of the same CEA sample seen in (A). The section in B) was incubated with 10-9 M [111In]In-DOTA-JR11, the section in C) was incubated with 10-9 M [111In]In-DOTA-JR11 plus a blocking dose of 10-6 M unlabelled DOTA-JR11. The inset shows the boxed region at higher magnification, the red arrows indicate sectioning artefacts. D) SST2 and E) CD68 immunohistochemistry on 5 µm sections adjacent to B) and C), with matching insets. Scale bar indicates two mm.
Copyright American Society of Nuclear Cardiology

## Slide 6
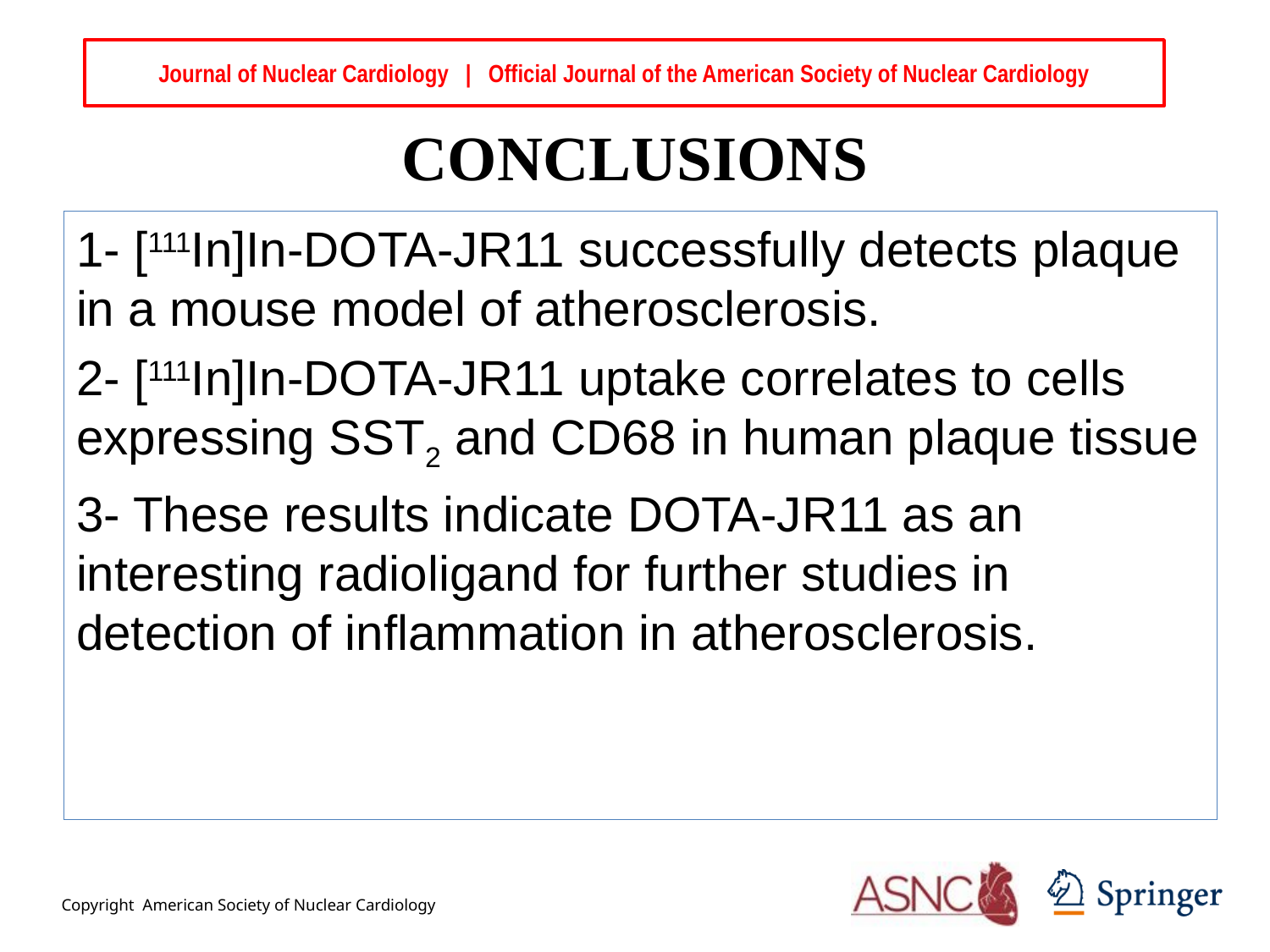

Journal of Nuclear Cardiology | Official Journal of the American Society of Nuclear Cardiology
# CONCLUSIONS
1- [111In]In-DOTA-JR11 successfully detects plaque in a mouse model of atherosclerosis.
2- [111In]In-DOTA-JR11 uptake correlates to cells expressing SST2 and CD68 in human plaque tissue
3- These results indicate DOTA-JR11 as an interesting radioligand for further studies in detection of inflammation in atherosclerosis.
Copyright American Society of Nuclear Cardiology
